# A Novel CCT5 Missense Variant Associated with Early Onset Motor Neuropathy

**DOI:** 10.3390/ijms21207631

**Published:** 2020-10-15

**Authors:** Vincenzo Antona, Federica Scalia, Elisa Giorgio, Francesca C. Radio, Alfredo Brusco, Massimiliano Oliveri, Giovanni Corsello, Fabrizio Lo Celso, Maria Vadalà, Everly Conway de Macario, Alberto J. L. Macario, Francesco Cappello, Mario Giuffrè

**Affiliations:** 1 Department of Health Promotion, Mother and Child Care, Internal Medicine and Medical Specialties, University of Palermo, 90127 Palermo, Italy; vincenzoantona@virgilio.it (V.A.); giovanni.corsello@unipa.it (G.C.); mario.giuffre@unipa.it (M.G.); 2 Department of Biomedicine, Neuroscience and Advanced Diagnostics (BIND), University of Palermo, 90127 Palermo, Italy; scalia.fede@gmail.com (F.S.); maria.vadala@unipa.it (M.V.); 3 Department of Biomolecular Strategies, Genetics and Advanced Therapies, Euro-Mediterranean Institute of Science and Technology (IEMEST), 90139 Palermo, Italy; AJLMacario@som.umaryland.edu; 4 Department of Medical Sciences, University of Torino, 10126 Torino, Italy; elisa.giorgio@unito.it (E.G.); brusco.alfredo@gmail.com (A.B.); 5 Genetics and Rare Diseases Research Division, Ospedale Pediatrico Bambino Gesù IRCSS, 00146 Rome, Italy; fclementina.radio@opbg.net; 6 Department of Psychological, Pedagogical and Educational Sciences, University of Palermo, 90128 Palermo, Italy; massimiliano.oliveri@unipa.it; 7 Department of Physics and Chemistry—Emilio Segrè, University of Palermo, 90128 Palermo, Italy; fabrizio.locelso@unipa.it; 8 Ionic Liquids Laboratory, Institute of Structure of Matter, Italian National Research Council (ISM-CNR), 00133 Rome, Italy; 9 Department of Microbiology and Immunology, School of Medicine, University of Maryland at Baltimore-Institute of Marine and Environmental Technology (IMET), Baltimore, MD 21202, USA; econwaydemacario@som.umaryland.edu

**Keywords:** CCT5, chaperonins, chaperoning system, genetic variants, mutation, genetic chaperonopathies, motor neuropathy

## Abstract

Diseases associated with acquired or genetic defects in members of the chaperoning system (CS) are increasingly found and have been collectively termed chaperonopathies. Illustrative instances of genetic chaperonopathies involve the genes for chaperonins of Groups I (e.g., Heat shock protein 60, *Hsp60*) and II (e.g., Chaperonin Containing T-Complex polypeptide 1, *CCT*). Examples of the former are hypomyelinating leukodystrophy 4 (HLD4 or MitCHAP60) and hereditary spastic paraplegia (SPG13). A distal sensory mutilating neuropathy has been linked to a mutation [p.(His147Arg)] in subunit 5 of the *CCT5* gene. Here, we describe a new possibly pathogenic variant [p.(Leu224Val)] of the same subunit but with a different phenotype. This yet undescribed disease affects a girl with early onset demyelinating neuropathy and a severe motor disability. By whole exome sequencing (WES), we identified a homozygous *CCT5* c.670C>G p.(Leu224Val) variant in the *CCT5* gene. In silico 3D-structure analysis and bioinformatics indicated that this variant could undergo abnormal conformation and could be pathogenic. We compared the patient’s clinical, neurophysiological and laboratory data with those from patients carrying p.(His147Arg) in the equatorial domain. Our patient presented signs and symptoms absent in the p.(His147Arg) cases. Molecular dynamics simulation and modelling showed that the Leu224Val mutation that occurs in the CCT5 intermediate domain near the apical domain induces a conformational change in the latter. Noteworthy is the striking difference between the phenotypes putatively linked to mutations in the same CCT subunit but located in different structural domains, offering a unique opportunity for elucidating their distinctive roles in health and disease

## 1. Introduction

Molecular chaperones (chaperones in short) are important components of all living cells in which they play a variety of roles. Canonical functions include assisting protein folding and refolding, translocation of proteins through membranes, ushering proteins damaged beyond repair to degradation, dissolution of protein aggregates, and selective autophagy. Chaperones perform protein quality control checks, essential for the maintenance of protein homeostasis [1]. Many chaperones belong to the category of “heat shock proteins” (Hsps), although not all Hsps are chaperones, and not all chaperones are Hsps (these terms are often used interchangeably). Chaperones are classified following several criteria, the most useful being their apparent molecular weight. Chaperones usually work in multimeric complexes, forming homo- or hetero-oligomeric teams, which associate with other molecules to build functional networks [1].

The term chaperonin is applied to members of a set of 55–64 kDa chaperones represented in humans by two groups: The mitochondrial Hsp60 (Cpn60, Group I) and the cytosolic chaperonin containing TCP-1 subunit (CCT, Group II).

A structurally and/or functionally defective chaperone is often associated with diseases, collectively termed “chaperonopathies”. Most of the hereditary chaperonopathies studied thus far tend to appear early in life and affect various tissues and organs, such as the central and peripheral nervous systems and the cardiocirculatory apparatus [2].

Chaperonopathies can be divided into categories, based on the chaperone gene-protein involved. Chaperonopathies due to mutations in small-size chaperones (sHsps) involve genes encoding chaperones with a MW 34 kDa or less, such as the crystallin subgroup; tissues affected are the eye lens, voluntary muscles, and peripheral nerves. Chaperonopathies due to mutations in the chaperonin genes involve *Hsp60* (Cpn60), which causes hereditary spastic paraplegia (SPG13, MIM605280) and MitCHAP-60 disease (Pelizaeus-Merzbacher-like, MIM612233). In Group II, *MKKS* causes the McKusick-Kaufman syndrome characterized by genitourinary malformations (MIM#236700), and *BBS10*, and *BBS12* Bardet-Biedl syndromes (BBS) type 10 (MIM # 615987) and 12 (MIM # 615989) [1]. A *CCT5* gene mutation is associated with a human phenotype, i.e., a mutilating, distal sensory neuropathy with severe atrophy of the posterior tract of the spinal cord [3,4]. Diseases associated with mutations in the genes encoding proteins within the Hsp40 and Hsp70 families, as well in the gene encoding the very large chaperone, sacsin have been described associated with a variety of neurologic disorders [2,5].

Here, we report a novel c.670C>G p.(Leu224Val) variant in *CCT5* that is likely associated with a disease characterized by early onset demyelinating motor neuropathy, expanding the range of phenotypes associated with this gene [3,4].

## 2. Results

### 2.1. Clinical History

**Figure 1:**
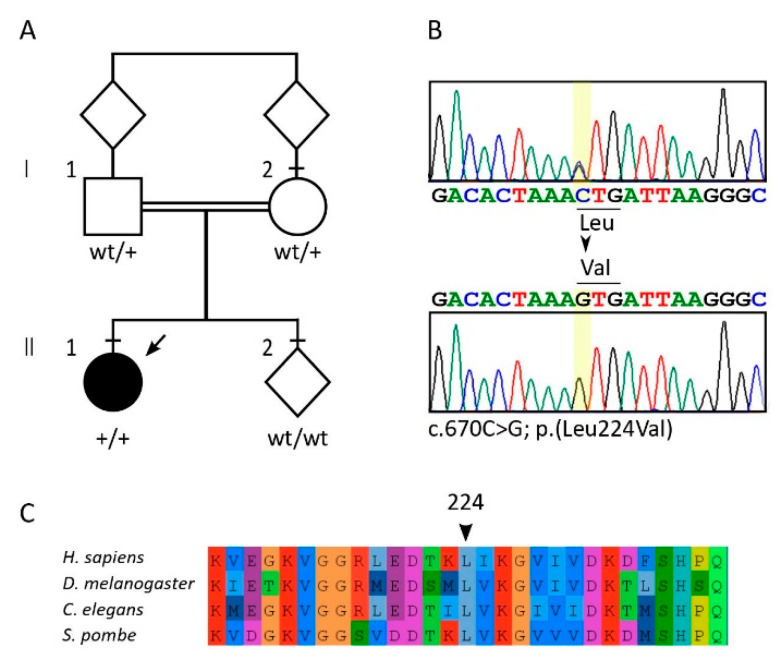
Pedigree (**A**) and genetic analyses (**B**). Whole exome sequencing identified a homozygous missense variant in exon 5 of *CCT5* c.670C>G p.(Leu224Val) (NM_012073). Leucine 224 is evolutionarily conserved from yeasts to vertebrates (**C**), each color corresponds to a single specific amino acid.

The proband was a macrosomic female newborn (birthweight 4500 g, 100th centile; length 52 cm, 93rd centile) born at term with eutocic delivery and good neonatal adaptation after an uneventful pregnancy. She was the first child of consanguineous first cousins (Figure 1A) with a positive family history for metabolic disorders.

A second cousin died at two years of age probably due to mitochondrial disease. Until eight months of age, she had a slow but otherwise normal neuro-psychomotor development. Later, her parents noted progressive hypotonia, developmental arrest and regression of sensorimotor and cognitive abilities. She presented difficulties getting to an upright position and was unable to walk without support showing a general progressive hyposthenia. After a few months, she presented with dystonic crises with extrapyramidal hypertonia, dystonic posturing of the extremities, fine tremors, and up-rolling of the eyes suggestive of seizures.

Electro-encephalogram (EEG) revealed a disorganized brain electrical activity, with theta and delta activity on left temporo-occipital derivations, and an asymmetric pattern consistent with the presence of clinical signs of generalized seizures. Brain magnetic resonance imaging (MRI) showed a mild delay of white matter myelination of the posterior regions. She was started on antiepileptic medications (clonazepam and trihexyphenidyl) and rehabilitation treatment with only partial response.

At two years of age, she was still unable to walk, to sit unsupported, to roll over, or voluntarily control sphincter function. She showed subsequent progressive worsening of dystonic crises (carbamazepine and haloperidol were included in the therapeutic scheme), neuronal impairment and muscular involvement with severe hypotonia and feeding problems (poor suck, swallowing difficulties, gastroesophageal reflux) leading to failure to thrive and requiring surgical management (Nissen fundoplication and PEG placement) at three years of age. Expressive language was absent. A prominent jaw dystonia hampered to fully close her mouth and produce normal speech. Intelligence quotient showed severe impairment. In the following months, a progressive worsening of dystonic crises was complicated by dyspnea, fever, and acute respiratory failure requiring intubation and mechanical ventilation. A failure to wean from mechanical ventilation led to tracheostomy at three and half years of age. Several hospital admissions followed, because of recurrent respiratory crises, worsening of hypotonia, severe respiratory acidosis, electrolyte disorders and anemia. Levetiracetam and phenobarbital have been introduced in the therapeutic scheme with poor results.

Main metabolic investigations gave normal results: organic acids, carnitine, congenital glycosylation disorders, very long chain fatty acid (VLCFA), full plasma amino acid profile, purine and pyrimidine, serum lactate, ammonia, glycemia, beta galactosidase, and transaminases (Table S1).

EEGs at four years of age revealed disorganization of bioelectrical activity, irregular background rhythm characterized by slow diffuse waves, high voltage delta waves prevalent in posterior head regions, absence of physiological figures of sleep, presence of rapid pharmacological activity. Chest X rays and orthopedic consultancy revealed lumbar spine scoliosis, related to severe hypotonia and postural effect. Brain MRI revealed thin corpus callosum, signs of ventricular enlargement and a pattern of diffuse hyperintensity of cerebral white matter, consistent with diffuse hypo/demyelination, enlargement of sulci and subarachnoid spaces mainly in the frontal and parietal areas.

We first met the patient at four years of age; her weight was 15 kg (25th centile), her length 93 cm (third centile) and her head circumference 47.5 cm (third centile). She presented with microcephaly, severe psychomotor delay and hypotonia, absence of language and any other expressive patterns, absence of sphincter function control.

**Figure 2:**
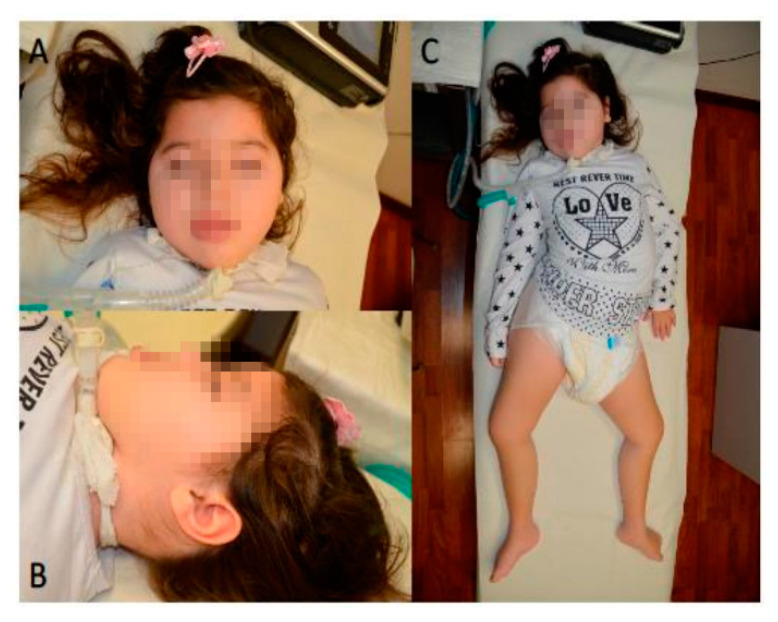
Proband at seven years of age showing microcephaly without evident dysmorphic features (**A**,**B**), tetra paresis and severe hypotonia (**C**).

Follow-up at seven years of age confirmed the growth pattern (30th centile for weight, sixth centile for length, second centile for head circumference) and the clinical picture of severe psychomotor delay and hypotonia without evidence of specific dysmorphic features (Figure 2; Table 1 and Table 2, and Table S1 (with: biochemical data for both mutations).

### 2.2. Neurological Examination and Electrophysiological Findings

At seven years of age, neurological examination revealed absent eye contact, horizontal nystagmus with episodic up-rolling of the eyes, tetraparesis, bilateral foot drop, marked axial and segmental hypotonia, incomplete head control and need to sit with support (Table 1 and Table 2). Osteotendinous reflexes were absent. Bilateral clonus of the foot was elicited following brisk stretching of the ankle. The clonus was sporadically accompanied by stiffening of the arms for 1–2 s.

### 2.3. Motor Evoked Potentials

Motor evoked potentials were absent following cortical stimulation in either the APB or TA muscles, Table 3. This pattern is consistent with impairment of the motor system, without a clear distinction between central and peripheral nervous system impairments.

### 2.4. Motor Nerve Conduction Study

Motor nerve conduction studies of the median and peroneal nerves showed signs of axonal and demyelinating neuropathy, with the presence of reduced motor amplitude and delayed motor latency responses after stimulation of distal sites of the median and peroneal nerves, Table 3. No compound motor evoked potentials were elicited by stimulation of proximal nerve sites.

### 2.5. Laboratory Investigations and Genetic Analyses

All routine laboratory biochemical investigations and metabolic studies were normal (full plasma amino acid profile, serum lactate, and ammonia). Cardiac, renal, hepatic, and urogenital ultrasound investigations were normal. Karyotype and array-CGH analysis performed on peripheral blood leukocytes were normal.

WES analysis identified 20 homozygous variants with an allele frequency equal to that found among controls < 0.01. Ten of these had a CADD score > 15. We further studied these genes, excluding eight variants associated with a class 2 or 3 ACMG (likely benign or VUS) in genes which are not associated to any known phenotype. A homozygous variant in *KIF*7, associated with Acrocallosal syndrome/Joubert syndrome 12 (MIM # 200990) was excluded because it is classified as likely benign. We also found the homozygous c.670C>G; p.(Leu224Val) in *CCT5* (NM_012073), which qualified as the only possible pathogenic variant by prioritization (Table S2, Figure 1).

Genotyping of maternal and paternal DNAs confirmed the variant at the heterozygous state in both, according to the recessive pattern of inheritance (Figure 1). CCT5 c.670C>G is reported at a low allele frequency among controls compatible with a rare recessive disease (5/251,400, rs925661175; GnomAD ver 2.1.1). Bioinformatics analyses showed the variant is predicted as deleterious by the majority of softwares (83% in Varsome, aug 2020), with a global domain-adversarial neural network (DANN) score of 0.9953, and a combined annotation-dependent depletion (CADD) score of 23.0 (PMID 25338716, 30371827.) The leucine 224 is highly conserved in evolution: the Vertebrate Multiz Alignment & Conservation (100 Species) track at UCSC genome browser (https://genome-euro.ucsc.edu/index.html) shows a complete identity among available vertebrate species. Sequence alignment of distantly related organisms up to *S. pombe* confirms a high conservation (Figure 1), supporting a key role for this amino acid in this position in protein function. Nevertheless, based on the rules of the American College of Medical Genetics and Genomics (ACMG) [6], the c.670C>G p.(Leu224Val) was classified as of uncertain significance.

### 2.6. Molecular Dynamics Simulation and Modelling

**Figure 3:**
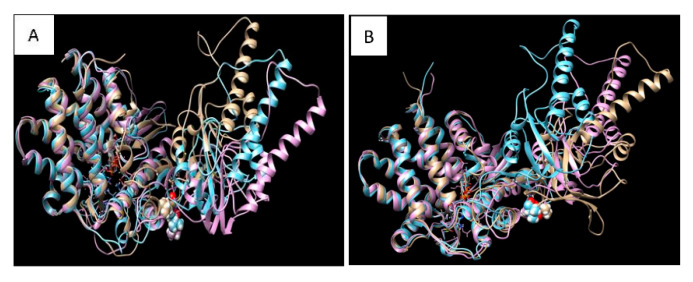
Protein conformational status for wild type and mutant CCT5 proteins. Image (**A**) is the superposition of the most probable conformations obtained from the molecular dynamics simulation of wild type CCT5 without nucleotide (gold), or bound to ATP (lilac), or bound to ADP (cyan). The image shows that the protein takes different conformations in the three conditions, especially at the level of the apical domain. Image (**B**) is the superposition of the most probable conformations obtained from the molecular dynamics simulation of mutant Leu224Val CCT5 without nucleotide (gold), or bound to ATP (lilac), or bound to ADP (cyan). The image shows that the mutant protein takes different conformations as compared with the wild type in the three conditions examined, especially at the level of the apical domain.

To further assess the impact of the mutation on the structure-conformation of the CCT5 subunit, molecular dynamic simulation was carried out with the wild-type and the mutant CCT5 molecules. They were compared under three forms: without ATP, with ATP, or with ADP (Figure 3).

The three forms were different for the wild-type molecule (Figure 3A) and for the mutant (Figure 3B), and those of the latter were differed from those of the wild type, particularly the nucleotide-free form. The apical domain of the nucleotide-free form of the mutant appears more relaxed than its counterpart in the wild type. It is noteworthy that the apical domain changed the most, although, the affected amino acid is in the intermediate domain.

## 3. Discussion

We describe a new disease affecting an Italian girl and characterized by early onset of severe progressive neuropathy, daughter of consanguineous first-degree relatives. Trio-WES revealed an homozygous c.670C>G p.(Leu224Val) missense variant in *CCT5*, which may play a pathogenic role. Although the variant is formally classified as of uncertain significance, bioinformatics analyses suggested a deleterious effect. The amino acid Leu224 is conserved not only in all vertebrates, but also in distantly related species such as *Drosophila, Caenorhabditis*, and even *Saccharomyces*, supporting the possibility of pathogenicity in its absence. Furthermore, in silico three-dimensional (3D) protein structure analysis suggested that the Leu224Val change could affect the protein’s conformation. The Leu224Val is located in the intermediate domain of the CCT5 subunit, but molecular dynamics simulations suggest that the mutation may have an effect on the structure of the apical domain, which could cause a global conformational alteration as it was observed for other mutations [7].

CCT functions as a hetero-oligomeric complex that consists of eight different subunits arranged into octameric rings with a central cavity, which pair by their open sides to form hexadecamers with a central chamber for protein folding [8,9]. The subunits are encoded by independent but evolutionarily related genes [10]. It has been estimated that as much as 10% of cytosolic proteins interact with the eukaryotic chaperonin CCT along their folding pathways [11]. CCT is required for the folding of essential proteins, including cytoskeletal proteins like tubulin and actin, as well as cell-cycle regulators such as CDC20, and CDH1 [9,12]. Each CCT subunit consists of three structural domains: a) apical, for substrate binding; b) equatorial, with ATP binding functions; and c) intermediate, for connecting the other two domains. The CCT5 subunit has a specific function within the CCT complex: it hydrolyzes ATP, working together with other neighboring subunits as a driver of energy for the whole complex [8].

The c.492A>G p.(His147Arg) variant in *CCT5* has previously been described in four siblings of a consanguineous Moroccan family showing an autosomal recessive mutilating sensory neuropathy with spastic paraplegia [3,4]. This disease, and the one described here, possibly associated with the variant p.(Leu224Val) share several neurological features, but also present peculiar differences that may be attributed to the different effects of the two variants and/or other genetic and acquired factors not yet identified.

Patients with the p.(His147Arg) have a severely impaired sensory function, especially affecting lower limbs. This led to disfiguring lower-limb lesions and ultimately amputation. Our pro-band does not show any sign of impaired sensory function, and no ulcerations are present in the upper or lower limbs, nor in any other part of the body. In all patients with the p.(His147Arg) variant, motor function was preserved, also in adulthood, when the patients were still autonomous in their movements. Furthermore, they did not show any deterioration in cognitive and coordination functions. On the other hand, our case showed significant motor impairments since the first year of life, which impeded to reach the standing position or to walk. Currently, at seven years of age, she is not autonomous in movements, respiration, and swallowing, and she is unable to communicate.

MRI revealed in patients with p.(His147Arg) an impaired myelination confined to the spinal cord; EEGs were not available. In our pro-band, hypomyelination and demyelination signs are more generalized as are the clinical signs and symptoms.

The skeletal system was normal in patients with p.(His147Arg); chest X-ray examination was not done. Our case presents lumbar spinal scoliosis, which could be a direct effect of the disease and/or the consequence of the severe hypotonia and predominant incorrect posture of a bedridden patient.

Patients with p.(His147Arg) had several biochemical abnormal parameters (hypotriglyceridemia, hypocholesterolemia, and low levels of ApoB lipoprotein) but they are within the normal ranges in our case (Table 1, Table 2 and Table S1).

Overall, all CCT5 p.(His147Arg) and p.(Leu224Val) cases present a neurologic disorder with central nervous system myelin involvement. From a clinical point of view, the p.(His147Arg) appears to be less aggressive and debilitating, involving mainly the sensory function. On the other hand, the p.(Leu224Val) shows an early onset motor neuropathy with a progressive severe impairment. This new variant could support the role for *CCT5* in neurologic disorders and suggest that a wider phenotypical spectrum of clinical manifestations may be associated with this gene.

## 4. Materials and Methods

### 4.1. Patients

The proband belongs to a large survey of pediatric syndromic cases collected at the Department of Health Promotion, Mother and Child Care, Internal Medicine and Medical Specialties, University of Palermo. Informed consent was collected from legal tutors (parents).

### 4.2. Motor Evoked Potentials

The patient was evaluated neurophysiologically with transcranial magnetic stimulation (TMS), recording motor evoked potentials from muscles of the upper (i.e., abductor pollicis brevis (APB) muscle) and lower (i.e., tibialis anterior (TA) muscle) limbs. A MagStim Rapid2 magnetic stimulator, connected with a figure of eight coil of 70 mm diameter was used to measure motor-evoked potentials. For upper limb recordings, the coil was positioned over a left scalp position corresponding to C3 site of the 10–20 EEG system. For lower limb recordings, the coil was positioned over a scalp position located 3 cm anterior to the Cz site of the 10–20 EEG system. Surface electrodes were applied with a belly-tendon montage on the APB muscle and on TA muscle. TMS intensity was gradually increased until the maximal intensity of the stimulator was reached.

### 4.3. Motor Nerve Conduction Study

The right median nerve was stimulated supra maximally at the wrist and elbow, while the right peroneal nerve was stimulated supra maximally at the ankle and head of the fibula through surface electrodes. Compound muscle action potentials (CMAPs) were recorded through surface electrodes placed over the APB muscle for the median nerve stimulation and over the extensor digitorum brevis (EDB) muscle for the peroneal nerve stimulation. The CMAPs were amplified (frequency range 20 Hz–10 kHz) and latencies and amplitudes were measured off-line. The distal motor latency from wrist to APB and from the ankle to EDB was measured and the motor nerve conduction velocity from elbow to wrist and from fibula to ankle were calculated.

### 4.4. Sequencing

The family provided written informed consent for the molecular analyses. Whole exome sequencing (WES) on the TRIO (proband, mother, and father) was performed using genomic DNA extracted from circulating peripheral blood leukocytes, Figure 2. WES data processing, sequence alignment to GRCh37, variant filtering and prioritization by allele frequency, predicted functional impact, and inheritance models were performed using an in-house implemented pipeline, which mainly takes advantage of the Genome Analysis Toolkit (GATK v.3.7), as previously described [6,7,8,9,10,11,12,13]. High-quality variants with an effect on the coding sequence or affecting splice site regions were filtered against public databases (dbSNP150 and gnomAD V.2.0) to retain; (i) private and clinically associated variants; and (ii) annotated variants with an unknown frequency or having minor allele frequency < 0.1%, and occurring with a frequency < 2% in an in-house database including frequency data from >1500 population-matched WES. The functional impact of variants was analyzed by CADD V.1.3, Mendelian Clinically Applicable Pathogenicity V.1.0 [14,15], and using InterVar V.0.1.6 to obtain clinical interpretation according to American College of Medical Genetics and Genomics/Association for Molecular Pathology 2015 guidelines [16].

Based on consanguinity of the asymptomatic parents who were first degree cousins, we assumed an autosomal recessive model of inheritance for the trait, and retained all the homozygous variants located within LoH genomic stretches using Homozygosity Mapper [17] (http://www.homozygositymapper.org), setting 80 as the number of consecutive homozygous SNPs. Variants were then filtered according to their predicted functional impact, retaining those variants with CADD score > 15 [14,18], and then prioritized considering the biological and clinical relevance of individual genes (HPO terms: Intellectual disability, Microcephaly, Seizures, Muscular hypotonia). Sequence validation were performed by Sanger sequencing using an ABI 3130XL and the ABI BigDye Terminator Sequencing Kit V 3.1, and sequences were examined using the SeqScape v2.6 Software (Applied Biosystems, Foster City CA 94404 USA).

### 4.5. Molecular Dynamics Simulation and Modelling

The structural properties of the CCT5 subunit, wild type and mutated, were obtained starting from the structure of the crystallized protein deposited in the Protein Data Bank with accession codes 5UYZ [19]. The structure of the mutant subunit was obtained by changing amino acid residue 224 Leucine with Valine, using the package Maestro Schrödinger LLC, New York, NY, 2018, version 11.6.010. Molecular dynamics (MD) simulations were performed for 150 ns (in some cases extended to 200ns), using the GROMACS 2018.3 package [20,21]. Interactions were described using an all-atoms CHARMM27 force field [22,23].

MD simulations for the various molecules and conditions were performed using a cubic box of NaCl 150 mM in explicit TIP3P water solution. Periodic boundary conditions were applied. The force field parameter files and initial configuration for the protein were created by GROMACS utilities programs. The equilibration procedure was performed in several steps, starting from an NVT (substance (N), volume (V) and temperature (T)) simulation at 300 K with the protein heavy atom positions restrained to equilibrate the solvent around it, followed by a NPT (substance (N), pressure (P) and temperature (T)) run at 300 K and pressure at 1 bar, for a 10 ns run. After the equilibration phase, the system was run for a total of 150 ns (200 ns) for an NVT production run; the trajectory was saved at a frequency of 10 ps to evaluate dynamical and structural properties. The MD simulations were always checked versus the root mean square displacement (RMSD) and the energy profile (data not reported). During the production runs a velocity rescaling thermostat [24] was used for the temperature coupling, with a time coupling constant of 0.1 ps. A Parrinello–Rahman barostat [25] was used for the pressure coupling, with relaxation constant of 1 ps. The equations of motion were integrated through the Leap-Frog algorithm, using a 2 fs time step.

The values of cut-off of the Lennard-Jones and real space part of the Coulombic interactions were set to 10 Å. The Particle Mesh Ewald (PME) summation method [26,27] was used to evaluate the electrostatic interactions, with an interpolation order of 4 and 0.16 nm of FFT (Fast Fourier Transform) grid spacing. The structures shown have been selected by clustering analysis [28,29,30] performed by the g cluster tool implemented in GROMACS package, following the method outlined in [31]. Selected images and protein manipulation were done using Maestro (Maestro, Schrödinger, version 11.6.010) and CHIMERA [32]. All the simulations have been carried out on a workstation equipped with Intel core I7 processor, 32 GB DDR3 system memory and Nvidia GeForce GTX 1080 TI GPU with 11GB DDR5 memory.

## 5. Conclusions

Here, we describe a patient affected by early onset, demyelinating motor neuropathy, and the presence of a homozygous variant c.670 C>G; p.(Leu224Val) in the gene encoding the subunit 5 of the chaperonin CCT. Whether the mutation is the only cause of the disease in our patient, or a causative factor among others, cannot be fully ascertained. Nevertheless, the Leu224Val variant seems to play a pathogenic role because it is the only genetic abnormality we could detect that could have the effects observed. Furthermore, the Leu224 is highly conserved in evolution, suggesting that it has a vital role and that its change will have a serious negative impact on cell physiology. In silico analyses showed that the mutation could induce conformational changes in the CCT5 molecule. If another family with the same mutation is discovered, more data will become available for clarifying the role of this variant. Meanwhile, the doors are open to continue research aiming at elucidating the impact of the Leu224Val variant on the intrinsic properties and functions of the encoded chaperonin subunit and its role, if any, in the mechanisms underpinning the tissue and organ lesions observed. In this regard, the availability of another mutation, on the same CCT5 subunit but on a different structural domain and associated with a different phenotype, offers a unique opportunity to elucidate the distinctive functions of the various parts of a single chaperone molecule in health and in chaperonopathies.

## Figures and Tables

**Table 1:** Clinical data for CCT5 variants.

	Leu224Val	His147Arg ^1^
**Gender; Age**	Female; 8 years.	Males; 34, 22, 19, and 7 years.
**Age at onset**	Eight months of age.	Early infancy, range from 1 to 5 years.
**Major clinical signs and symptoms**	Progressive hypotonia and hyposthenia. Absent osteotendinous reflexes. Bilateral clonus of the foot. Dystonic crisis with extrapyramidal hypertonia, dystonic posturing of the extremities, fine tremors, and up-rolling of the eyes suggestive of seizures. Absent eye contact, horizontal nystagmus, tetra paresis, marked axial and segmental hypotonia, incomplete head control.	Spasticity in walking with legs in form of X. Increased tendon reflexes with ankle clonus, Babinsky sign, and discreet distal amyotrophy. Normal motor function. Sensory loss for all modalities in both upper and lower limbs, particularly in foot. Normal cranial nerves, coordination, and cognitive functions.
**General disease progression**	Progressive worsening of dystonic crisis with neuronal impairment. Severe hypotonia and feeding problems (poor suck, swallowing difficulties, gastroesophageal reflux). Difficulties getting an upright position and inability to walk. Dyspnea, fever, and acute respiratory failure. Absence of sphincter function control. Expressive language absent. Jaw dystonia. Severe impairment of intelligence quotient.	Oldest patient: scars of healed ulcers in hands and osteomyelitis of the feet leading to amputation of his two legs up to the thighs.

^1^ Data from references [3,4] (Bouhouche et al., 2006a,b).

**Table 2:** Neurophysiological and laboratory data for both mutations.

Data Source	Leu224Val	His147Arg ^1^
MRI ^2^	Mild delay of white matter myelination of the posterior regions. Thin corpus callosum, signs of ventricular enlargement and a pattern of diffuse hyperintensity of cerebral white matter, consistent with diffuse hypo/demyelination, enlargement of sulci and subarachnoid spaces mainly in the frontal and parietal areas.	Severe atrophy of the spinal cord predominantly in the posterior tract.
EEG	Disorganized brain electrical activity, with theta and delta activity on left temporo-occipital derivations. Asymmetric pattern (generalized seizures). Irregular background rhythm characterized by slow diffuse waves; high voltage delta waves prevalent in posterior head regions. Absence of physiological figures of sleep. Presence of rapid pharmacological activity.	Not reported
Motor nerve conduction studies (see also Table 3)	Signs of both axonal and demyelinating motor neuropathy upon stimulation of the median and peroneal nerve. Presence of reduced motor amplitude and delayed motor latency responses after stimulation of distal sites of the median and peroneal nerves. No compound motor evoked potentials were elicited by stimulation of proximal nerve sites.	Normal or slightly reduced motor conduction velocity of the median and the peroneal nerves.
Motor evoked potentials (see also Table 3)	Absent motor evoked potentials following cortical stimulation in either the APB or TA muscles.	Not reported
Ultrasound scanning	Normal cardiac, renal, hepatic, and urogenital ultrasound results.	Not reported.
X-rays	Chest X rays, lumbar spine scoliosis.	Not reported
Karyotype	Normal	Not reported
Array CGH	Normal	Not reported
Biochemical measurements (see also Table S1).	Normal levels of Apo B lipoprotein, total cholesterol, and triglycerides. Other: normal values of organic acids, carnitine, congenital glycosylation disorders, VLCFA, full plasma amino acid profile, purine and pyrimidine, serum lactate, ammonia, glycemia, beta galactosidase and transaminases.	Decreased levels of Apo B lipoprotein, total cholesterol, and triglycerides. Other: not reported.

^1^ Data from references [3,4] (Bouhouche et al., 2006a,b). ^2^ Abbreviations: MRI, Magnetic resonance imaging; EEG, electroencephalogram; APB, abductor pollicis brevis; TA, tibialis anterior; CGH, comparative genomic hybridization (for microarray-based comparative genomic hybridization).

**Table 3:** Transcranial magnetic stimulation and motor nerve conduction studies for the Leu224Val.

**Transcranial magnetic stimulation**	**Target Muscle**		**Latency**			**Amplitude**	
	APB ^1^		Absent Response			Absent Response	
	TA		Absent Response			Absent Response	
**Nerve conduction study**	**Nerve**	**Distal Latency (ms)**		**Proximal Latency (ms)**	**Distal Amplitude (mV)**		**Proximal Amplitude (mV)**
	median	5.1		absent response	0.9		0
	peroneal	7.2		absent response	1.2		0

^1^ Abbreviations: APB, abductor pollicis brevis; TA, tibialis anterior.

## Supplementary Materials

The following are available online at https://www.mdpi.com/1422-0067/21/20/7631/s1, Table S1: Biochemical data for both mutations. Table S2: Homozygous variants in proband.
