# Supplementary material for: A Novel CCT5 Missense Variant Associated with Early Onset Motor Neuropathy
Source: Int J Mol Sci. 2020 Oct 15;21(20):7631. doi: 10.3390/ijms21207631 (PMC7589105; doi:10.3390/ijms21207631)
Supplement: Supplementary file 1 [file ijms-21-07631-s001.zip › ijms-951527-supplementary.docx]

**Table 1. Biochemical data for both mutations.**

| **Analyte^1^** | **Normal range** | **Leu224Val** | **His147Arg^2^** |
| --- | --- | --- | --- |
| Glycaemia, mg/dL | 60-100 | 115 | Not reported |
| Lactic acid, mg/dL | 8-16 | 19.50 |  |
| Ammonium, ug/dL | < 75 | 36 |  |
| BUN, mg/dL | 5-18 | 11 |  |
| Creatinine, mg/dL | 0.3-0.7 | 0.4 |  |
| Uric acid, mg/dL | 1.7-5.8 | 1.2 |  |
| Alkaline Phosphatase, IU/L | 140-200 | 86 |  |
| hs-CRP mg/dL | < 0.5 | 2.35 |  |
| gamma GT, IU/L | 5-32 | 18 |  |
| Apo A lipoprotein, mg/dl | 110-160 |  | 125 |
| Apo B lipoprotein, mg/dL | 60-150 | 140 | 39 |
| TC mg/dL | 140-200 | 177 | 121 |
| TG mg/dL | 50-180 | 59 | 33 |

^1^Abbreviations: BUN, blood urea nitrogen; hs-CRP, high sensitivity C-reactive protein; TC, total cholesterol; TG, triglycerides.

^2^Data from references 3 and 4 (Bouhouche et al., 2006a, b).

**Table 2.** Homozygous variants in proband.

| **#CHROM** | **POS** | **GENE** | **Feature_ID** | **Var_nt_pos** | **Var_aa_pos** | **Effect** | **Disease_name** | **Inheritance** | **MIM #** | **CADD** | **Varsome (ACMG class)** | **GnomAD AF** | **GnomAD HOM** |
| --- | --- | --- | --- | --- | --- | --- | --- | --- | --- | --- | --- | --- | --- |
| chr2 | 112988507 | ZC3H8 | NM_032494.2 | c.859_863delAAGAA | p.Lys287fs | frameshift_variant |  |  |  | 34 | class 3 | 0.004049 | 2 |
| chr2 | 129025851 | HS6ST1 | NM_004807.2 | c.1121G>A | p.Ser374Asn | missense_variant | Hypogonadotropic hypogonadism 15 with or without anosmia | AD | 614880 | 12.17 | class 2 | 0.0008421 | 0 |
| chr2 | 135095831 | MGAT5 | NM_002410.4 | c.647C>T | p.Ala216Val | missense_variant |  |  |  | 24.7 | class 2 | 0.00005911 | 0 |
| chr4 | 1388788 | CRIPAK | NM_175918.3 | c.517_518insGACGTGGAGTGCCCGCCTGCTCACACGTGCCCATGTGGAGTGCCCGCCTGCTCACACGTGCC | p.Pro173fs | frameshift_variant |  |  |  | 22.9 | na | 0.0002901 | 0 |
| **chr5** | **10258362** | **CCT5** |  | **c.670C>G** | **p.Leu224Val** | **missense_variant** | **Early onset, demyelinating, motor neuropathy (putative)** | **AR** |  | **24.7** | **class 3** | **0.00001989** | **0** |
| chr5 | 140166929 | PCDHA1 | NM_018900.3 | c.1054G>C | p.Val352Leu | missense_variant |  |  |  | 0.001 | class 3 | 0 | 0 |
| chr5 | 149215873 | PPARGC1B | NM_133263.3 | c.1855C>A | p.Pro619Thr | missense_variant |  |  |  | 16.31 | class 2 | 0.000004026 | 0 |
| chr5 | 149264117 | PDE6A | NM_000440.2 | c.1952A>G | p.Asn651Ser | missense_variant | Retinitis pigmentosa 43 | AR | 613810 | 10.93 | class 3 | 0.001094 | 0 |
| chr5 | 159839530 | SLU7 | NM_006425.4 | c.571-15_571-5dupTTTTTTTTTTT | . | splice_region_variant |  |  |  | . | class 2 | 0.006454 | 0 |
| chr6 | 159188550 | EZR | NM_001111077.1 | c.1345-6C>T | . | splice_region_variant |  |  |  | 5.163 | class 3 | 0 | 0 |
| chr6 | 160230182 | PNLDC1 | NM_001271862.1 | c.775G>A | p.Ala259Thr | missense_variant |  |  |  | 24.6 | class 3 | 0.00001195 | 0 |
| chr6 | 160499402 | IGF2R | NM_000876.3 | c.5478+8G>T | . | splice_region_variant |  |  |  | 0.372 | class 3 | 0.000003988 | 0 |
| chr11 | 118774002 | BCL9L | NM_182557.2 | c.680_691delGGGGCGGGGGCG | p.Gly227_Gly230del | disruptive_inframe_deletion |  |  |  | 17.81 | class 3 | 0.001097 | 1 |
| chr11 | 119058667 | PDZD3 | NM_001168468.1 | c.676C>A | p.Gln226Lys | missense_variant |  |  |  | 16.15 | class 3 | 0 | 0 |
| chr14 | 94733364 | PPP4R4 | NM_058237.1 | c.2447C>T | p.Ala816Val | missense_variant |  |  |  | 17.18 | class 3 | 0.0005211 | 0 |
| chr14 | 96904221 | AK7 | NM_152327.3 | c.659C>T | p.Ala220Val | missense_variant | ?Spermatogenic failure 27 | AR | 617965 | 13.34 | class 3 | 0.0006129 | 0 |
| chr15 | 29561014 | NSMCE3 | NM_138704.3 | c.896G>T | p.Gly299Val | missense_variant | Lung disease, immunodeficiency, and chromosome breakage syndrome | AR | 617241 | 7.741 | class 2 | 0.000004004 | 0 |
| chr15 | 54307871 | UNC13C | NM_001080534.2 | c.2771G>A | p.Gly924Asp | missense_variant |  |  |  | 11.49 | class 2 | 0.0003965 | 1 |
| chr15 | 90196025 | KIF7 | NM_198525.2 | c.137G>A | p.Arg46His | missense_variant | Acrocallosal syndrome/Joubert syndrome 12 | AR | 209900 | 24.4 | class 2 | 0 | 0 |
| chr19 | 44337636 | ZNF283 | NM_181845.1 | c.1-5_1-4dupTT | . | splice_region_variant |  |  |  | . | class 3 | 0 | 0 |

Note: Homozygous variants with a AF < 0.01 are shown. Variants with a CADD score >15 are highlighted in yellow.
